# Leverson on Vaccination

**Published:** 1897-08

**Authors:** J. W. Thomson

**Affiliations:** 159 West Forty-eighth St.; New York


### LEVERSON ON VACCINATION.

NEW YORK, June 25th, 1897.

TO THE EDITOR OF THE HOMŒOPATHIC PHYSICIAN.

Dr. M. R. Leverson, whose articles on the burning question of vaccination have much interested me, asks if I will write to encourage you to continue their publication.

Little as you may need such encouragement, I yet do so with pleasure. I believe vaccination to be radically wrong, therefore always injurious. His exhaustive statistical work, from the best sources, will, I believe, be unanswerable as to the fallacies of vaccination. But that is not the question. Every one has the inalienable right to adopt any mode of treatment that may seem best to him, but has no right to force it upon any one else. It is against *compulsory* vaccination that we declaim, as an interference with human liberty and legalized outrage and crime. Anything that in any way exposes the nefariousness of this crime is useful and timely. It is supported by fraud, misrepresentation, and intimidation. The poor suffer most. The doctors have police accompany them to make their wholesale and indiscriminate vaccinations. In Brooklyn the police forcibly held a woman down while the myrmidons of the so-called Board of Health vaccinated her. Think of it! That such proceedings are possible. The true physician should be welcome, "as the flowers in May." He comes to heal. Where healing is impossible, to soothe and succor. To make the inevitable path to the grave, which all must tread, lose its terrors, as much as may be. Such an one needs no executive of law and civil force. His mission is his credentials. The knowledge of the healing blessing he brings, his welcome. It is especially incumbent upon the Hahnemannian to fight against medical force. The whole history of Homœopathy is interwoven with its struggle against craft, coercion, and lies. While this was especially marked in its early history, it is equally true to-day. Its professed friends who masquerade in its borrowed garments are its worst enemies. You, Doctor, were for many years associated with the venerable Lippe, one of the most sturdy and fearless advocates of the truth. We may place him side by side with the illustrious and renowned Hahnemann, in his unflinching advocacy of our cause. They knew no fear and gave no favors in their fight for the truth. Personal considerations were of no moment. Their lives gave the lie to the blatant assertion that every man has his price.

The need of the hour is to fight as these men fought, against compulsory vaccination. There can be no rest until this insult to freedom is wiped off the statutes of our government.

159 West Forty-eighth St. J. W. Thomson, M. D.

